# Characteristics and treatment results of recurrence in adult-type granulosa cell tumor of ovary

**DOI:** 10.1186/s13048-020-00619-6

**Published:** 2020-02-14

**Authors:** Dan Zhao, Yanan Zhang, Zhengjie Ou, Rong Zhang, Shan Zheng, Bin Li

**Affiliations:** 1 Department of Gynecology Oncology, National Cancer Center/ National Clinical Research Center for Cancer/Cancer Hospital, Chinese Academy of Medical Sciences and Peking Union Medical College, No.17, South Panjiayuan Residential, Chaoyang District, Beijing, 100021 China; 2 Department of Pathology, National Cancer Center/ National Clinical Research Center for Cancer/Cancer Hospital, Chinese Academy of Medical Sciences and Peking Union Medical College, No.17, South Panjiayuan Residential, Chaoyang District, Beijing, 100021 China

**Keywords:** Ovarian granulosa cell tumor, Adult granulosa cell tumor, Recurrent granulosa cell tumor, Prognosis

## Abstract

**Background:**

The aim of this study was to explore the clinicopathological characteristics of recurrent adult-type granulosa cell tumor of the ovary (AGCOT) and evaluated the treatment results to define the prognostic parameters for survival after recurrence.

**Results:**

A retrospective review of 40 patients with recurrent AGCOT, who were treated in the Cancer Hospital at the Chinese Academy of Medical Sciences from 2000 to 2015 was conducted. The impact of clinical and pathological characteristics, progression-free survival (PFS), and post-recurrence therapeutic approaches on prognosis were analyzed. Among the 40 recurrent patients, there were 10 cases where the relapse was uncontrolled, 24 cases had second relapses, and 6 cases without further relapses at the time of our follow-up. The median PFS was 61 months (range, 7-408 months), and the median time interval between the first and the second relapses (R-PFS) was 25 months (range, 0–94 months). The median time interval between the first relapse and death (R-OS) was 90 months (range, 2–216 months). PFS ≥ 61 months (*P* = 0.004) and post-recurrence therapeutic approach (*P* < 0.001) were independent risk factors for repeated recurrences. The age at recurrence (*P* = 0.031) and post-recurrence therapeutic approach (*P* = 0.001) were independent risk factors for death after recurrence.

**Conclusion:**

Among patients with recurrent AGCOT, those with long PFS had good prognoses. Maximal cytoreductive effort should be made after recurrence. Complete resection and postoperative adjuvant chemotherapy may improve the prognosis of patients with recurrent AGCOT.

## Background

Ovarian granulosa cell tumor accounts for 2–3% of all ovarian cancer patients [1]. According to the 2014 World Health Organization histological classification of ovarian tumors, ovarian granulosa cell tumors are divided into two types: ovarian adult granulosa cell tumor (AGCOT) and ovarian juvenile granulosa cell tumor (JGCOT) [2]. Ovarian adult granulosa cell tumor, accounting for 95% of ovarian granulosa cell tumor, mainly occurs in perimenopausal and postmenopausal women and exhibits unpredictably late recurrence features [3–6]. Among the multiple prognostic factors reported, menopause, tumor diameter, estrogen and CA125 levels, International Federation of Gynecology and Obstetrics (FIGO) staging, residual lesions, multisite recurrence, and presence of mitotic figures have been mentioned as factors associated with the prognosis [7–10]. The treatment and outcomes of recurrent AGCOT remain uncertain, as there have only been a small number of case reports [11–15]. The present study focuses on clinicopathologic parameters and treatment results in recurrent adult granulosa cell tumor of the ovary.

## Materials and methods

This present study included 40 cases of recurrent AGCOT treated in the Cancer Hospital at the Chinese Academy of Medical Science from 2000 to 2015, including 7 cases who received the initial treatment in our hospital and 33 cases who received the initial treatment in other hospitals. All patients had follow-ups until May 2019. Recurrence was recorded only in patients proven to be tumor-free after initial treatment (i.e., having no residual tumor after surgery or chemotherapy, the latter being dependent upon a clean computed tomography [CT] scan). The recurrent site was judged by a CT scan or during cytoreductive surgery, and multisite recurrence was defined as tumors found at more than two anatomical regions. Initial and post-recurrent clinical features and therapeutic approaches—including age, menopause status, surgical approaches, tumor staging, chemotherapy status, recurrent site, post-recurrence therapeutic approaches—were reviewed from the medical records. Histopathological sections of surgical specimens were retrieved and reviewed by a senior pathologist to record and analyze tumor necrosis, atypia, hemorrhage, mitotic figures, and immunohistochemical biomarkers. The prognosis-related data were obtained through telephone interviews and outpatient follow-ups. SPSS 23.0 software (IBM SPSS., Chicago, IL) were used for data analysis. The influencing factors of recurrent progression-free survival (R-PFS) and recurrent overall survival (R-OS) were analyzed by the Kaplan-Meier method, and the comparison was performed using the log-rank method. The risk factors of second recurrence were analyzed by univariate and multivariate analyses using the Cox proportional hazard regression. A *P* < 0.05 was considered as a statistically significant difference. The research protocol of this study was approved by the Ethics Committees of the National Cancer Center/Cancer Hospital at the Chinese Academy of Medical Sciences.

## Results

### Patient and tumor characteristics at initial diagnosis

The age of initial disease onset was 21–62 years, with a median age of 44.5 years. There were 14 cases of menopause at the initial treatment and 26 cases without menopause. Among the 40 cases, 37 patients had given birth, and 3 patients had never given birth. The common clinical manifestations were abdominal pain/distension, a pelvic mass, and vaginal bleeding. There were 10 cases of abdominal pain and distension, 20 cases of a pelvic mass, and nine cases of vaginal bleeding. All cases underwent surgery as the initial treatment. Fourteen cases underwent fertility-preservation surgical procedures, including three cases of cystectomy, eight cases of unilateral adnexectomy, and three cases of unilateral adnexectomy and omentectomy. The remaining 26 cases had a hysterectomy and bilateral salpingo-oophorectomy (HBSO), as well as staging and cytoreductive surgery. Of all patients, 18 cases were staged and 22 cases were not staged. Five patients underwent lymphadenectomy, and 35 patients had no lymphadenectomy. For FIGO staging, there were 30 cases of stage I, including 3 cases of stage Ia, 15 cases of stage Ic, and 12 cases of stage Ix (either stage Ia or Ic); there were 5 cases of stage II and 5 cases of stage III. Among all patients, 11 cases had no postoperative chemotherapy, and 29 cases had postoperative chemotherapy. Of the cases receiving postoperative chemotherapy, there were 8 cases of BEP regimen, 9 cases of TC/ paclitaxel plus cisplatin (TP) regimen, and 12 cases of other regimens. Sixteen patients received chemotherapy for four cycles or less, and 13 patients received chemotherapy for more than four cycles (Table 1).

**Table 1:** Patient and tumor characteristics at initial diagnosis (*N* = 40)

Characteristics	n (*N* = 40)	%
Age	21–62	44.5 (median)
Place of initial treatment		
Our hospital	7	17.5%
Other hospital	33	82.5%
Parturition		
Yes	37	92.5%
No	3	7.5%
Menopause		
Postmenopausal	14	35%
Premenopausal	26	65%
Stage		
I	30	75%
II	5	12.5%
IIIc	5	12.5%
Side		
Unilateral	38	95%
Bilateral	2	5%
surgery at initial diagnosis		
Fertility preserve	14	35%
No preserve	26	65%
Staging		
Yes	18	45%
No	22	55%
Lymphadenectomy		
Yes	5	12.5%
No	35	87.5%
Adjuvant chemotherapy		
No	11	27.5%
Yes	29	72.5%
Chemotherapy regimen(*N* = 29)		
BEP	8	27.6%
TC/TP	9	31.0%
Other	12	41.4%

### Clinicopathological features and therapeutic approaches at recurrence

**Fig. 1:**
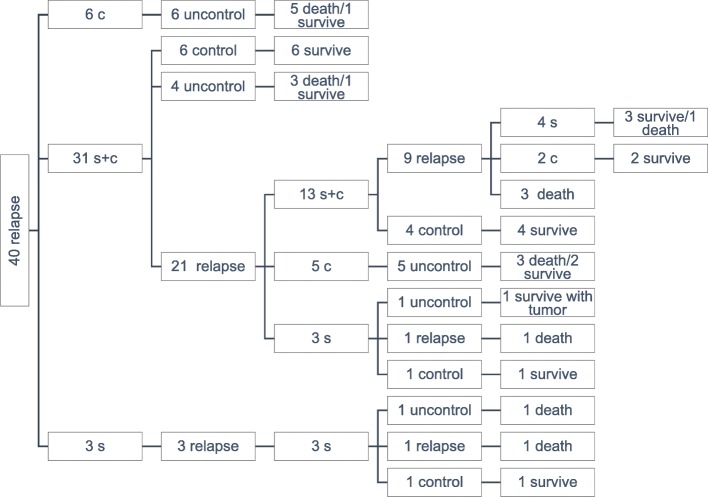
Clinical outcomes of 40 patients with recurrent AGCOT, including 18 deaths and 22 survival cases. Abbreviations: s, surgery; c, chemotherapy

Among the 40 patients with recurrence, the PFS was 7–408 months and the median was 61 months; the OS was 34–493 months, with a median of 126 months. The age at recurrence ranged from 22 to 77 years, with a median of 50.6 years old. There were 19 cases that had clinical symptoms, while 16 cases were asymptomatic in which the recurrence was discovered by imaging examination during follow-ups. The other five cases had no record of whether they had symptoms. The recurrence sites included pelvic recurrence in 15 cases, multisite recurrences in the abdominopelvic cavity in 24 cases, and lung metastasis with pleural effusion in 1 case. Eight patients had recurrence of a single lesion, and 32 patients had recurrence of multiple lesions. Among the cases in which the recurrent lesions involved the abdominal cavity, seven cases involved the greater omentum; five cases involved the liver; three cases involved the perisplenic region; four cases involved the abdominal wall. Among the patients with recurrence, the post-recurrence therapeutic approaches included 3 cases of surgery alone, 6 cases of chemotherapy alone, and 31 cases of surgery combined with adjuvant chemotherapy. Twenty-four had second recurrences and 11 had third recurrences. A total of 18 deaths were reported (Fig. 1). Among the 34 patients with post-recurrence surgery, 23 patients underwent complete resection of the lesion, and 11 patients had residual lesions after the surgery. Among the 31 patients with post-recurrence chemotherapy, 9 patients underwent a BEP regimen; 16 patients underwent paclitaxel plus platinum-based regimens; 6 patients underwent other chemotherapeutic regimens. Ten patients had ≤ four-cycles chemotherapy, and 21 patients had > four-cycles chemotherapy. Reviewing of the pathological reports of the 40 patients with recurrence showed that 20 patients had inhibin-A examination, including 18 positive cases (+ − +++) and 2 negative cases. Eleven patients had vimentin examination, including only 1 negative case and 10 positive cases (+ − +++). Eleven patients had an S-100 protein examination, including 10 positive cases (+ − +++) and 1 negative case; 15 patients had a Ki-67 protein examination, with only 1 case was 60% and the rest were below 30%. Among the 22 cases with histopathological sections of post-recurrence surgical specimens being retrieved and reviewed by a senior pathologist, 4 patients had necrosis and 18 patients did not have necrosis. There were 10 cases of mild atypia, 11 cases of moderate atypia, and 1 case of severe atypia. There were 20 cases of hemorrhage and 2 cases without hemorrhage There were 6 cases of > five mitotic figures/HPF and 16 cases of < 5 mitotic figures/HPF (Table 2).

**Table 2:** Patient and tumor characteristics at recurrence(*N* = 40)

Characteristics	n (*N* = 40)	%
Age	22–77	50.6 (median)
Clinical symptoms at recurrence		
Yes	19	47.5%
No	16	40%
Unknown	5	12.5%
Recurrence site		
Pelvic	15	37.5%
Abdominal/ abdominopelvic cavity	24	60%
Distant (lung metastasis)	1	2.5%
Uni/multisite Recurrence		
Uni-site	8	20%
Multi-site	32	80%
Treatment		
Surgery	3	7.5%
Chemotherapy	6	15%
Surgery + chemotherapy	31	77.5%
Complete resection(*N* = 34)		
No	11	32.4%
yes	23	67.6%
Necrosis (*N* = 22)		
No	18	81.8%
Yes	4	18.2%
Atypia		
Mild	10	45.5%
Median	11	50%
Severe	1	4.5%
Mitotic		
< 5	16	72.7%
> 5	6	27.3%
Prognosis		
Re-recurrence	24	60%
Uncontrol	10	25%
Control	6	15%
Death	18	45%

### Analysis of the influencing factors of R-PFS and R-OS

Until the last follow-up in May 2019, the follow-up duration of the 40 patients with recurrence was 57–298 months, with a median follow-up of 134 months. The R-PFS was defined as the time between the start of the post-recurrence treatment and the repeated recurrence or disease progression in the patient, or between the start of the post-recurrence treatment and the time of the last follow-up of the patients with no progressive disease. The R-OS was defined as the time from the start of the post-recurrence treatment until the death of the patients or the last follow-up. Our results showed that the R-PFS of the patients ranged from 0 to 94 months, with a median R-PFS of 25 months. The R-OS of the patients ranged from 2 to 216 months, with a median R-OS of 90 months. The three-year survival rate after recurrence was 82.4%, and the five-year survival rate after recurrence was 76.6%.

Univariate analysis of recurrence features and the effect of post-recurrence therapeutic approaches on the prognosis of recurrent AGCOT showed that PFS (*P* = 0.014), post-recurrence therapeutic approach (*P*< 0.001), and post-recurrence atypia of surgical pathology (*P* = 0.030) had significant impacts on R-PFS. Additionally, post-recurrence therapeutic approach (*P* = 0.002) and complete resection after recurrence (*P* = 0.003) had significant impacts on R-OS. Patients with PFS ≥ 61 months, post-recurrence surgery combined with adjuvant chemotherapy, and mild atypia had a relatively long R-PFS. Cox multivariate analysis—including the age at recurrence, PFS, single/multisite recurrence, and post-recurrence therapeutic approach—showed that the PFS ≥ 61 months and the post-recurrence therapeutic approach were independent risk factors for second recurrence.

**Fig. 2:**
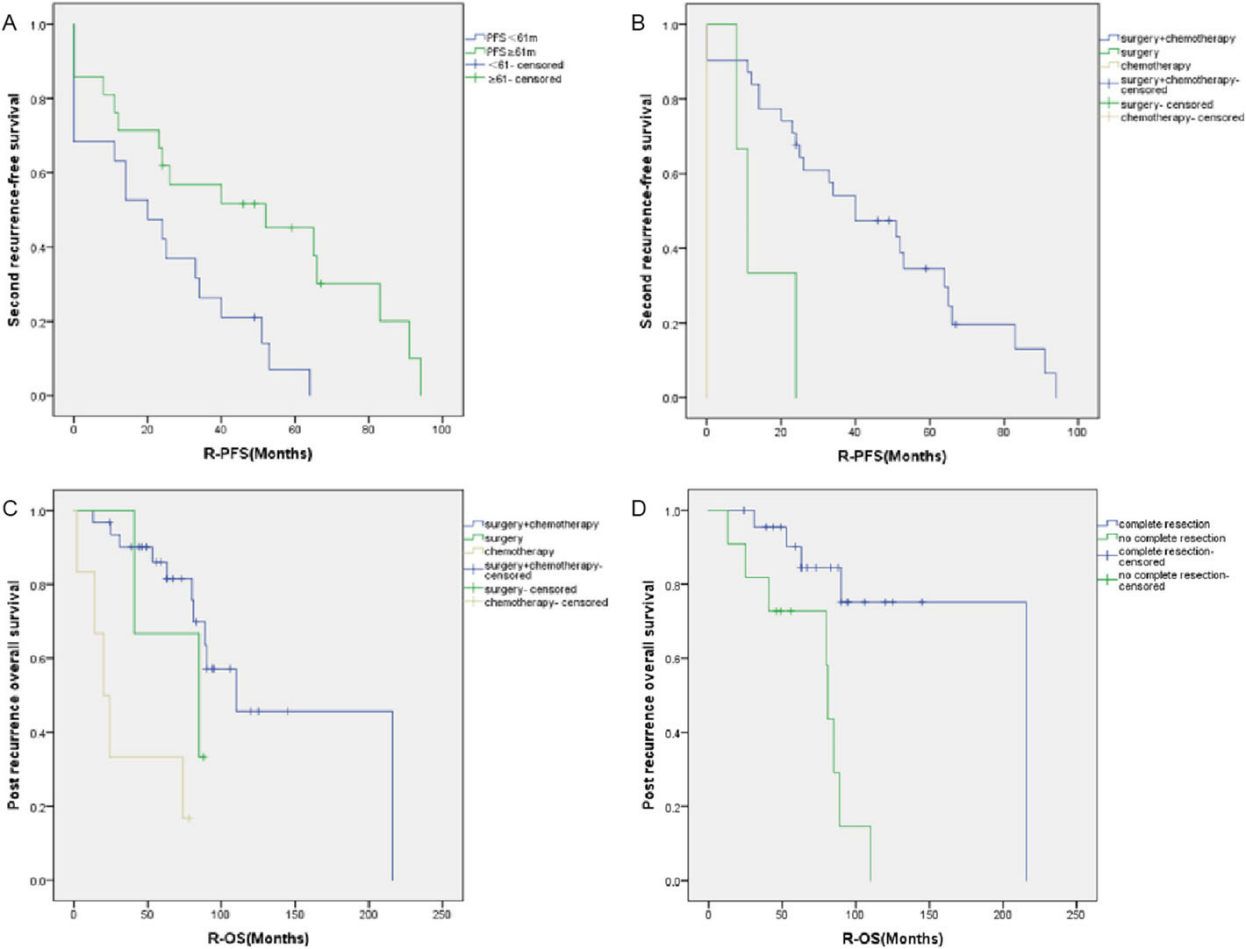
R-PFS according to PFS (**a**) and post-recurrence therapeutic approaches (**b**). R-OS according to post-recurrence therapeutic approaches (**c**) and post-recurrence complete resection (**d**). Abbreviations: R-PFS, Time interval between the first and the second relapses; c, chemotherapy; R-OS, Time interval between the first relapse and death; PFS, progression-free survival.

Patients with PFS < 61 months had a 3.5-fold higher risk of a second recurrence than patients with PFS ≥ 61 months. Patients with post-recurrence surgery alone and post-recurrence chemotherapy alone had 10.6-fold and 15-fold higher risks of a second recurrence, respectively, than those of patients with surgery combined with adjuvant chemotherapy. The age at recurrence > 50 years (*P* = 0.031) and post-recurrence therapeutic approaches (*P* = 0.001) were independent risk factors for post-recurrence death. Patients who were > 50 years old at recurrence had a 3.3-fold higher risk of death compared to that of patients who were < 50 years old at recurrence. Patients with post-recurrence chemotherapy alone had a 13.4-fold higher risk of death than that of patients with post-recurrence surgery combined with chemotherapy (Table 3). Figure 1 shows the outcome of the 40 patients with recurrent AGCOT. Effects of PFS and post-recurrence therapeutic approach on R-PFS, as well as effects of post-recurrence therapeutic approach and post-recurrence complete resection of lesions on R-OS, are presented in the survival curves in Fig. 2.

**Table 3:** Univariate and multivariate analysis after recurrence

Characteristics	Recurrence risk factors analysis				Death risk factors analysis			
	Univariate analysis		Multivariate analysis		Univariate analysis		Multivariate analysis	
	HR(95% CI)	*P*	HR(95% CI)	*P*	HR(95% CI)	*P*	HR(95% CI)	*P*
Age, y (*N* = 40)								
≤ 50	1	0.215			1	0.076	1	0.031
>50	1.553 (0.775–3.114)				2.439 (0.887–6.708)		3.331 (1.116–9.946)	
PFS (Median 61 m) (*N* = 40)								
≥ 61 m	1	0.014	1	0.004	1	0.321		
< 61 m	2.619 (1.217–5.639)		3.537 (1.503–8.327)		1.657 (0.611–4.492)			
Recurrence site (*N* = 40)								
Uni-recur	1	0.508			1	0.388		
Multi-recur	0.751 (0.321–1.755)				1.917 (0.437–8.409)			
therapy after recurrence (*N* = 40)								
Surgery+chemotherapy	1	0.000	1	0.000	1	0.002	1	0.001
Surgery	4.883 (1.318–18.083)	0.018	10.599 (2.472–45.441)	0.001	2.341 (0.483–11.345)	0.291	2.069 (0.426–10.047)	0.367
Chemotherapy	15.216 (3.576–64.750)	0.000	15.192 (3.589–64.307)	0.000	9.196 (2.650–31.908)	0.000	13.418 (3.410–52.793)	0.000
Complete resection (*N* = 34)			N/A	N/A			N/A	N/A
Yes	1	0.081			1	0.003		
No	2.147 (0.910–5.065)				6.324 (1.860–21.504)			
Chemotherapy regimens (*N* = 31)			N/A	N/A			N/A	N/A
BEP	1	0.076			1	0.709		
TC/TP/TN/TI	0.675 (0.266–1.714)	0.408			1.037 (0.204–5.264)	0.965		
Other	2.346 (0.795–6.924)	0.123			1.754 (0.390–7.885)	0.464		
Cycle (*N* = 31)			N/A	N/A			N/A	N/A
≤ 4	1	0.832			1	0.308		
>4	1.097 (0.467–2.574)				2.241 (0.475–10.570)			
Necrosis (*N* = 22)			N/A	N/A			N/A	N/A
No	1	0.109			1	0.596		
Yes	2.574 (0.809–8.190)				1.536 (0.315–7.481)			
Atypia (*N* = 22)			N/A	N/A			N/A	N/A
Mild	1	0.030			1	1.000		
Median	2.676 (0.976–7.337)	0.056			1.000 (0.267–3.742)	1.000		
Severe	19.720 (1.593–244.051)	0.020			1.000 (0.000–12,512.695)	1.000		
Mitotic (*N* = 22)			N/A	N/A			N/A	N/A
< 5	1	0.334			1	0.308		
> 5	1.639 (0.601–4.470)				2.074 (0.510–8.439)

Analysis of the 34 patients with post-recurrence surgery showed that PFS < 61 months and postoperative chemotherapy (*P* = 0.001) were independent risk factors for a second recurrence. Patients with PFS < 61 months had a 5.4-fold higher risk of a second recurrence than that of patients with PFS ≥ 61 months. Patients without complete resection of lesions had a 6.6-fold higher risk of death than that of patients with complete resection of lesions (*P* = 0.003; Table 4).

**Table 4:** Univariate and multivariate analysis of patients undergoing surgery after recurrence (*N* = 34)

Characteristics	recurrence risk factors analysis				death risk factors analysis			
	Univariate analysis		Multivariate analysis		Univariate analysis		Multivariate analysis	
(*N* = 34)	HR(95% CI)	*P*	HR(95% CI)	*P*	HR(95% CI)	*P*	HR(95% CI)	*P*
Age,y								
≤ 50	1	0.282			1	0.293		
>50	1.521 (0.708–3.267)				1.879 (0.580–6.084)			
PFS (Median 61 m)								
≥ 61 m	1	0.007	1	0.001	1	0.126		
< 61 m	3.327 (1.384–8.000)		5.439 (1.926–15.363)		2.625 (0.761–9.047)			
Complete resection								
Yes	1	0.081			1	0.003	1	0.003
No	2.147 (0.910–5.065)				6.324 (1.860–21.504)		6.586 (1.909–22.730)	
Chemotherapy								
No	1	0.018	1	0.001	1	0.284		
Yes	0.205 (0.055–0.759)		0.067 (0.014–0.320)		0.422 (0.087–2.044)			

## Discussion

Approximately 75% of patients with AGCOT are at stage I, with the lesion being confined to the ovary. Surgical resection is the main treatment for AGCOT and results in good prognosis. In the present study, 30 (75%) out of 40 patients with recurrent AGCOT were at stage I diagnosis. However, 20–25% of AGCOT patients would have recurrence and are characterized by long-term recurrence, with a median recurrence time of 4–6 years after the initial treatment, and as long as 40 years after the treatment [5]. Due to the low incidence and recurrence rates and long recurrence time of AGCOT, the therapeutic and prognostic analyses of post-recurrence cases have been relatively rare. Only a small number of recurrence cases have been reported [5, 8, 11, 13, 14, 16]. The largest report currently available is from a multicenter study in Taiwan, with a total of 44 cases of recurrence that had a median PFS and OS of 61.5 months and 115.3 months, respectively [15]. Dridi et al. reported that the average PFS was 8.4 years, and the average OS was 13 years [3]. Results of the present study are consistent with previous findings. As the largest single-center case study of recurrent AGCOT, the present study showed that 40 recurrent cases had a median PFS of 61 months and a median OS of 126 months, with the post-recurrence three-year survival rate of 82.4% and five-year survival rate of 76.6%. The PFS < 61 months was the independent risk factor of second recurrence. We speculate that this result may be due to patents with a short PFS being prone to developing drug resistance to platinum-based chemotherapy, thereby reducing the efficacy of post-recurrence chemotherapy. Patients with longer PFS have better sensitivity to repeated chemotherapy after recurrence. The PFS is an important factor in predicting the chemotherapy sensitivity and prognosis for patients with recurrent epithelial ovarian cancer [17], while the clinical value of the length of PFS in recurrent AGCOT has not been clarified. Because AGCOT is a tumor with low malignant potential, its overall sensitivity to chemotherapy is relatively poor. Although this point of view has not been reported in other studies, a longer PFS of patients suggests a better prognosis after recurrence and should be given active treatment in clinical practice.

AGCOT recurs at various stages and involves various parts of the abdominopelvic cavity that are similar to epithelial-derived ovarian malignant tumors. Abu-Rustum et al. [18] reported that pelvic recurrence accounted for 70% of AGCOT recurrence, pelvic and abomdinal recurrence accounted for 9%, retroperitoneal recurrence accounted for 6%, pelvic and retroperitoneal recurrence accounted for 6%, and pelvic, abdominal, and retroperitoneal recurrence accounted for 3%. According to the analysis of Fotopoulou et al. of the dissemination patterns of AGCOT and comparisons between recurrent AGCOT and initial lesions, recurrent AGCOT is prone to peritoneal dissemination (15.8% vs. 52%), middle abdominal cavity (15.8% vs. 48.1%), and upper abdominal metastasis (0 vs. 33.3%) [12]. A study by Lee et al. [13] has shown that recurrent AGCOT often occurs in the pelvis, followed by the liver and small intestine, and can even metastasize into lungs and bones. A study by Dridi [2] has shown that AGCOT mostly recurs in the pelvis, abdomin, and liver. In the present study, similar metastastic features were found in the 40 recurrent AGCOT cases, including 33 cases (82.5%) of recurrent lesions involving the pelvic cavity and 19 cases (47.5%) of recurrent lesions involving the abdominal cavity (including 7 in the greater omentum, 5 in the liver, 3 in the perisplenic region, and 4 in the abdominal wall incision). Among the 40 recurrent cases, 8 cases had single site recurrence, whereas 32 cases had multisite lesions. Therefore, our results indicate that the recurrence pattern of AGCOT consists of pelvic-based multisite metastasis. The greater omental and liver metastases were most common in the upper abdomen. However, the recurrent site and multifocality had no signifciant effect on the prognosis, which might be related to the relatively high complete resection rate of the repeated cytoreductive surgery in the patients with recurrent AGCOT.

Pelvic and paraaortic lympadenectomy have not been used as a routine surgical procedure for initial staged operations in patients with AGCOT, because lymph-node metastasis (LNM) of such patients is only 4.5–5.5% [19, 20]. Retroperitoneal LNM is more likely to occur in recurrent cases [14, 18]. Abu-Rustum et al. reported that up to 15% of patients with first recurrent AGCOT had a retroperitoneal LNM [18]. Brown et al. [21] reported that 6 of 117 (5%) patients with recurrent ovarian sex-cord-stromal tumors had LNM. Among the six patients, three had no lymph-node involvement and the remaining three patients had no lymph-node assessment performed at the time of initial surgery. This result suggests that despite an absence of LNM during initial treatment, LNM might still occur in the recurrence. Therefore, it is important to evaluate the status of retroperitoneal lymph nodes in patients with recurrent AGCOT, and lymphadenectomy should be actively performed in patients with swollen lymph nodes. In the present study, 5 of the 40 patients with recurrent AGCOT underwent lymphadenectomy at initial sugery, and no LNM was found. Only one had LNM at the time of recurrence and the patient was one of the five. The rate of LNM in AGCOT is low, and can still occur in patients who have undergone lymphadenectomy at the initial treatment. Therefore, full assessment of lymph-node status during recurrence is necessary to detect LNM.

Recurrent AGCOT involves multiple quadrants in the abdominopelvic cavity and multiple organs. Treatment of recurrent AGCOT is difficult and there are no standard therapeutic approaches. However, multiple therapeutic regimens—such as surgery, chemotherapy, radiotherapy, and endocrine therapy—are often comprehensively selected according to specific disease conditions. Few large studies related to post-recurrence therapeutic approaches have been published. Lee et al. proposed that active surgical treatment is an important regimen for primary and recurrent AGCOT [13]. Crew et al. hypothesized that cytoreductive surgery for complete resection of metastatic lesions is feasible, even though abdominopelvic metastasis occurs in recurrent AGCOT [22]. A retrospective study of 35 cases of recurrent AGCOT by Mangili et al. [14] showed that, among the five cases of liver metastasis (5/35), three patients had resection of metastatic lesions in the liver, and two patients survived without a tumor until the last follow-up. Moreover, 13 patients were relapsed among the 32 patients with complete resection of the initial surgery, while three patients with residual tumors all recurred. Six patients with residual tumors under secondary cytoreductive surgery all had a second recurrence, while only 11 out of 28 patients with complete resection had recurrence. These findings indicate that not only patients with residual tumors in initial surgery had increased risk of recurrence in newly treated AGCOT patients [13, 23], but that the presence of residual tumors during the secondary cytorective surgery also affected the prognosis of patients after recurrence. In the present study, among the 34 patients with post-recurrence surgery, patients with residual tumors had a 6.6-fold higher risk of death compared to that of patients without residual tumors. A study by Fotopoulou [12] et al. showed that compared with newly diagnosed patients (who could have nearly 100% complete resection in the cytoreductive surgery), 85% patients with recurrent AGCOT had their visible residual tumors completely removed, of which 33.3% of recurrent cases had the lesion affecting the upper abdomen. Although the rate of complete resection was slightly lower than that of the newly diagnosed cases, the 85% complete resection rate of the recurrent patients with multiple metastasis in the abdominopelvic cavity was much higher than that of malignant epithelium ovarian patients. This finding was associated with the low degree of malignancy and the lack of a large amount of ascites in the recurrent AGCOT patients, who had a better general conditions and surgical tolerance. In the current study, among the 34 patients with post-recurrence surgery, 23 patients (67.6%) underwent complete resection and 11 patients had residual lesions; the latter had multiple lesions involving the liver and spleen, which led to great challenge in surgical resection. The patients who got complete resection after recurrence had longer R-OS. Therefore, we believe that surgery is the most important therapeutic regimen for recurrent AGCOT, and that active multidisciplinary surgery should be performed as much as possible to achieve complete resection and to improve prognostic outcomes of the patients.

Chemotherapy can be used as a palliative treatment for inoperable patients with recurrent AGCOT or as a consolidation treatment after secondary cytoreductive surgery. However, the role of chemotherapy in the initial treatment of recurrent granulosa cell tumors of the ovary has been controversial. For AGCOT patients with initial treatment, patients receiving chemotherapy are mostly patients with stage Ic or above. Studies have shown that postoperative adjuvant chemotherapy does not improve the prognosis and does not prolong the PFS or OS of the patients [24, 25]. A retrospective study by Mangili et al. in 35 cases of recurrent AGCOT showed chemotherapy did not improve the prognosis of the patients with recurrence who underwent secondary cytoreductive surgery, and the author recommended the patients who had no residual tumor in the secondary cytoreductive surgery may omit adjuvant chemotherapy. In the present study, the percentage of the patients receiving platinum-based chemotherapy before or after the recurrence was 72.5 and 92.5% respectively. Among the 40 recurrent AGCOT cases, patients with surgery alone or chemotherapy alone had a significantly higher risk of recurrence and risk of death than those of patients with postoperative adjuvant chemotherapy. Among the 34 patients who received post-recurrence surgery, the risk of a second relapse in the 31 patients with postoperative adjuvant chemotherapy was significantly lower than that of the 3 patients with post-recurrence surgery alone, suggesting that postoperative adjuvant chemotherapy might improve the therapeutic outcomes of recurrent AGCOT patients with secondary cytoreductive surgery contrary to Mangili [14]. Chemotherapy could be used as a palliative treatment for recurrent AGCOT patients who are inoperable or who are unable to have a complete tumor resection. Post-recurrence chemotherapy regimens include BEP, paclitaxel plus platinum-based regimens (such as TC, TP, and paclitaxel plus nedaplatin [TN]), and paclitaxel plus ifosfamide (TI). A previous study has shown that TC or paclitaxel alone has a similar therapeutic effect as that of the BEP chemotherapy regimen; additionally, paclitaxel is less toxic [26]. In the present study, univariate analysis showed that postoperative chemotherapies and the number of courses of chemotherapy did not affect the R-PFS or R-OS in the 31 recurrent AGCOT patients, suggesting that paclitaxel plus platinum-based regimens and four treatment courses could be used as a choice for post-recurrence chemotherapy in recurrent AGCOT patients.

Among the pathological features of AGCOT, necrosis, mitotic figures, and atypia are relatively rare but are closely related to disease prognosis. Studies have shown that cellular atypia, a high mitotic index (4–10 mitoses per 10 HPF), and an absence of Call-Exner bodies are the only significant histologic predictors of early recurrence [27]. However, studies on pathological and immunohistochemical features in recurrent AGCOT patients have rarely been reported. In the present study, pathological sections of 22 patients (22/34) who had post-recurrence surgery were retrieved and reviewed by a senior pathologist and were all confirmed to be recurrent AGCOT. Like the newly diagnosed AGCOT, univariate analysis showed that patients with mild atypia (*P* = 0.030) were able to achieve longer R-PFS, suggesting that recurrent AGCOT patients with moderate and severe atypia were more likely to have second recurrence and should be given active comprehensive treatment.

AGCOT is a rare ovarian malignant tumor with slow growth and is prone to late recurrence. Given its broad time span, implementation of prospective studies of AGCOT is difficult. This retrospective study analyzed 40 recurrent AGCOT cases treated in our hospital from 2000 to 2015. This time period was chosen because the therapeutic approaches for AGCOT patients in our hospital were not uniform before 2000, which used a variety of surgical methods and chemotherapy programs. Therefore, this led to a bias in our case selection. Although this was a retrospective study with a small number of included cases, it represents the largest number of recurrent AGCOT cases from a single-center study since 2000. The present study focused on analyzing the clinicopathological features and prognostic factors in recurrent AGCOT in order to guide future individualized clinical treatments.

## Conclusion

AGCOT is an ovarian tumor with a low malignant potential that is prone to late recurrence and multiple recurrences. The prognosis of patients with a longer tumor-free interval before AGCOT recurrence was better. Maximal cytoreductive surgery is recommended. Complete resection combined with postoperative adjuvant chemotherapy may improve the prognosis of recurrent AGCOT.

## Data Availability

The datasets used and analyzed during the current study are available from the corresponding author on reasonable request.

## Abbreviations

AGCOT: Adult-type granulosa cell tumor of the ovary
BEP: Cisplatin etoposide and bleomycin
CA125: Carbohydrate antigen-125
CT: Computed tomography
FIGO: International Federation of Gynecology and Obstetrics
HBSO: Hysterectomy and bilateral salpingo-oophorectomy
HPF: High power field
JGCOT: Juvenile-type granulosa cell tumor of the ovary
LNM: Lymph node metastasis
PFS: Progression-free survival
R-OS: The median time interval between the first relapse and death
R-PFS: The median time interval between the first and the second relapses
TC: Paclitaxel plus carboplatin
TI: Paclitaxel plus ifosfamide
TN: Paclitaxel plus nedaplatin
TP: Paclitaxel plus cisplatin
